# Application of non-contact sensors for health monitoring in hospitals: a narrative review

**DOI:** 10.3389/fmed.2024.1421901

**Published:** 2024-06-12

**Authors:** Yoo Jin Choo, Gun Woo Lee, Jun Sung Moon, Min Cheol Chang

**Affiliations:** ^1^Department of Physical Medicine and Rehabilitation, College of Medicine, Yeungnam University, Daegu, Republic of Korea; ^2^Department of Orthopaedic Surgery, College of Medicine, Yeungnam University, Daegu, Republic of Korea; ^3^Division of Endocrinology and Metabolism, Department of Internal Medicine, College of Medicine, Yeungnam University, Daegu, Republic of Korea

**Keywords:** health monitoring, non-contact, sensor, hospital, review

## Abstract

The continuous monitoring of the health status of patients is essential for the effective monitoring of disease progression and the management of symptoms. Recently, health monitoring using non-contact sensors has gained interest. Therefore, this study aimed to investigate the use of non-contact sensors for health monitoring in hospital settings and evaluate their potential clinical applications. A comprehensive literature search was conducted using PubMed to identify relevant studies published up to February 26, 2024. The search terms included “hospital,” “monitoring,” “sensor,” and “non-contact.” Studies that used non-contact sensors to monitor health status in hospital settings were included in this review. Of the 38 search results, five studies met the inclusion criteria. The non-contact sensors described in the studies were radar, infrared, and microwave sensors. These non-contact sensors were used to obtain vital signs, such as respiratory rate, heart rate, and body temperature, and were then compared with the results from conventional measurement methods (polysomnography, nursing records, and electrocardiography). In all the included studies, non-contact sensors demonstrated a performance similar to that of conventional health-related parameter measurement methods. Non-contact sensors are expected to be a promising solution for health monitoring in hospital settings.

## 1 Introduction

Health monitoring is crucial in hospital settings for early detection and prevention of diseases as well as for assessing the effectiveness of treatments (1, 2). Regular monitoring of health status enables the early detection of disease onset or health-related issues and helps identify the risk of comorbidities and complications (3). Moreover, monitoring disease progression can help decelerate or prevent deterioration (4, 5). Health monitoring of patients being treated in hospitals can also help track patient recovery and increase treatment efficiency (6, 7). Furthermore, patients admitted to hospitals often experience post-hospital syndrome characterized by temporary frailty and an increased risk of readmission due to inactivity or sleep deprivation following admission (8). Monitoring of the heart rate (HR) and sleep can help reduce the incidence of post-hospital syndrome (8). Considering various clinical situations, hospital health monitoring is perceived as both important and essential.

Traditionally, contact-based devices (e.g., electrocardiogram recorders, continuous blood glucose monitoring devices, and respiratory belts) have been utilized in clinical settings for hospital-based health monitoring. Electrocardiograms measure vital signs via patches attached to the skin that receive electrical signals; however, they carry the risk of skin disorders owing to patch usage (9, 10). Invasive devices can also induce fear in patients (11), whereas wearable devices can cause inconvenience owing to difficulties in wearing the devices (12). Additionally, general vital sign monitors can induce psychological distress in patients owing to their bulky size (13). Moreover, the cables connecting the patient to the device can cause discomfort when the patients move (14, 15). Non-contact sensors have been developed to overcome the limitations of conventional devices in health monitoring. Through non-contact sensors, patients can monitor their health status in real-time without wearing specific equipment, thereby improving convenience and comfort in daily life (16, 17). Additionally, non-contact sensors pose a minimal risk of skin disorders because they do not directly touch the patient’s body and can reduce psychological burdens owing to their small size and inconspicuous nature (18, 19). Non-contact sensors can be particularly useful in hospital environments where the risk of infection is high. Hospitals are recognized as places where infection control is critical. Thus, non-contact sensing technology can play a significant role in reducing the spread of infections within hospitals by minimizing direct contact between patients and healthcare providers (20). In addition, non-contact sensing technology is known to be effective for quickly monitoring the health status of many patients. Non-contact thermometers or heart rate monitors can reduce the waiting time necessary for medical examinations and increase efficiency by reducing the workload of healthcare providers, as they do not require direct intervention. Several previous studies have demonstrated the performance of non-contact sensors in health monitoring. In 2019, Michler et al. (21) highlighted the usefulness of radar-based non-contact sensors in measuring the HR and respiratory rate (RR). In 2022, He et al. (22) reported that non-contact sensors based on depth cameras and radar could accurately detect the RR and respiratory patterns. Similarly, in 2022, Talukdar et al. (23) reported that a camera-based non-contact sensor using remote photoplethysmography (PPG) technology could be a novel solution for monitoring HR, RR, oxygen saturation, and blood pressure. Non-contact sensors in health monitoring appear to overcome the limitations of traditional methods and have the potential to be useful in hospital settings.

In this study, we compared the performance of traditional measuring devices with that of non-contact sensors for health monitoring in hospital settings. We conducted an in-depth examination of the current limitations of non-contact sensing technology and assessed the potential clinical applicability of non-contact sensors. In addition, by proposing future research directions, we aim to provide new perspectives in the fields of medicine and sensing technologies.

## 2 Methods

### 2.1 Search strategy

We searched the PubMed database for relevant studies published up to February 26, 2024. The following search terms were used: “hospital,” “monitoring,” “sensor,” and “non-contact.” The inclusion criteria for the studies were as follows: (1) studies of non-contact sensors used to monitor health status in hospitals and (2) studies that compared the performance of non-contact sensors and traditional methods for health monitoring. The exclusion criteria were as follows: (1) studies involving only healthy individuals; (2) studies involving wearable devices; (3) reviews, conference presentations, letters to the editor, or other unidentified types of articles; (4) studies published in languages other than English owing to the authors’ limited language abilities.

### 2.2 Data extraction

All search results were exported to Endnote 20 software. Two independent reviewers (Y. J. C. and M. C. C.) confirmed the retrieved studies and selected eligible ones. We checked the titles and abstracts to select studies that met the selection criteria and read the full texts to determine which articles were to be included in this study. Any disagreements between the reviewers were resolved through discussion.

## 3 Results

### 3.1 Selection of studies

**Figure 1:**
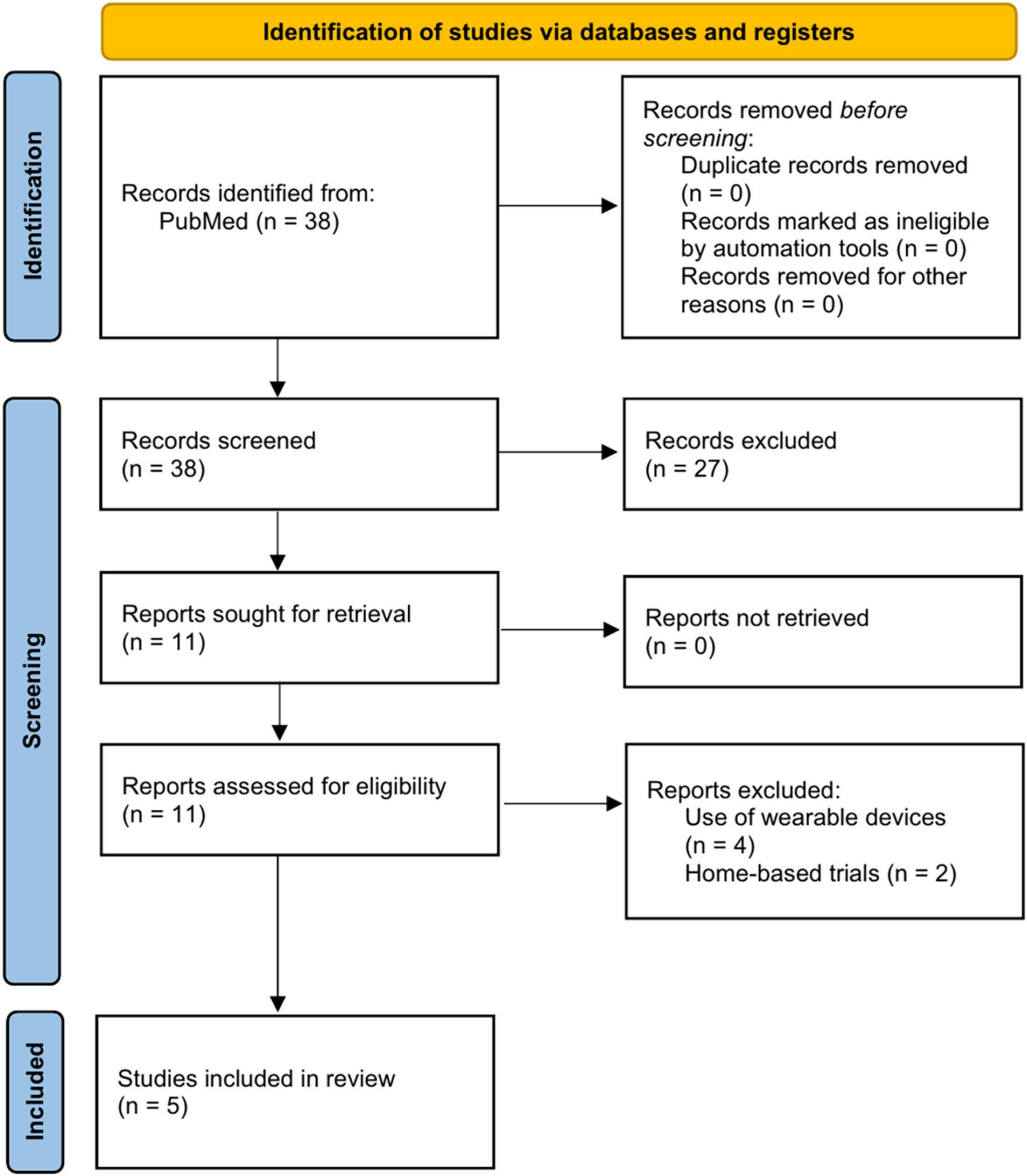
Flow chart for the selection of studies.

Of the 38 studies, 27 were excluded after checking the titles and abstracts of the studies that met the selection criteria. On checking the full texts of the remaining 11, four were excluded because they included wearable devices and two were excluded because they were conducted in a home environment. Finally, five studies (24–28) were included in this narrative review (Figure 1). The characteristics of the included studies are summarized in Table 1.

**Table 1:** Characteristics of selected studies.

Study	Participants	Target disorder	Non-contact sensor	Conventional measurement methods	Outcome parameters
Edanami et al. 2022 (24)	*N* = 3, mean age = 34 days	One patient with urinary tract infection and two patients with newborn jaundice	24 GHz radar (NJR4262; New Japan Radio Co., Ltd., Japan)	Electrocardiogram (ECG) bed monitor (BSM-6301; NIHON KOHDEN Co., Japan)	Heart rate, inter-beat interval, and heart rate variability
Tasi et al. 2020 (25)	*N* = 2, mean age = no information	Coronavirus disease	Self-injection locking radar (SIL Radar Technology INC., Kaohsiung, Taiwan)	Nursing record	Heart rate, repiratory rate, and body temperature
Weinreich et al. 2018 (26)	*N* = 57, mean age = 56.4 ± 14.0 years	Obstructive sleep apnea in 51 patients, Cheyne-Strokes respiration in six patients. Periodic limb movements of sleep in 19 out of 57 patients.	SleepMinder (BiancaMed, Dublin, Ireland)	Polysomnography	Sleep disorder index
Yoon et al. 2020 (27)	*N* = 10, mean age = no information, range of age = 40 to 80 years	Sleep disorders	Passive infrared sensor, a microwave sensor (WAVE, Sharp, Japan), and a smartphone (Galaxy Note 8; Samsung, Korea) application	Polysomnography	Sleep stages and sleep quality
Zaffaroni et al. 2009 (28)	*N* = 157, mean age = 53.9 ± 13.7 years	Sleep apnea	SleepMinder (BiancaMed, Dublin, Ireland)	Polysomnography	Apnea-hypopnea index

### 3.2 Summary of selected studies

In 2009, Zaffaroni et al. (28) compared the effectiveness of non-contact sensors (SleepMinder, BiancaMed, Dublin, Ireland) and polysomnography (PSG) in estimating the apnea-hypopnea index (AHI) of 157 participants with suspected sleep apnea. SleepMinder is a radar sensor-based non-contact sleep status monitoring device, developed to measure the breathing status and body movement during sleep. SleepMinder extracts respiratory signals from a patient’s chest movements and is typically placed facing the patient’s upper body because it has directionality that allows it to measure movement only in front of the sensor (29). In addition, it has a limited range and can only respond to objects within 2.5 meters of the sensor (29). The operating principle of SleepMinder is similar to that of the Doppler effect, a phenomenon in which the wave frequency changes depending on the relative motion of the wave source (26). SleepMinder detects and analyzes the phase shifts occurring in moving objects, such as breathing. Radiofrequency energy at 5.8 GHz was transmitted as two short pulses, each 5 ns long (30). The two pulses serve as the main transmit pulse, which is reflected from the object and received by the sensor, and the mixer pulse, which generates a signal proportional to the phase change of the main transmit pulse inside the receiver. SleepMinder operates at a low power with an average radiated power of 0.25 mW, meeting the safety and regulatory guidelines for radiofrequency devices (29). PSG is considered the gold standard for diagnosing sleep disorders (31). In PSG, apnea is generally defined as the complete cessation of breathing or a decrease in airflow of 90% or more lasting for more than 10 s (32). Hypopnea is typically defined as a 50% or greater decrease in airflow or a 3% or greater decrease in oxygen saturation lasting >10 s (32). AHI is a scale that determines the severity of sleep apnea and is classified into normal (AHI < 5), mild (5 ≤ AHI < 15), moderate (15 ≤ AHI < 30), and severe (AHI ≥ 30) (28). In Zaffaroni et al.’s study (28), SleepMinder was installed along with PSG to record biomotion signals simultaneously. The correlation coefficient between AHI estimates from SleepMinder and PSG was 0.91, indicating a strong positive correlation.

In 2018, Weinreich et al. (26) evaluated the performance of SleepMinder in detecting obstructive sleep apnea (OSA) and periodic limb movements during sleep (PLMS). Fifty-seven patients with sleep disorders, including 19 with PLMS, participated in this study. The levels of OSA and PLMS are typically determined using the AHI and periodic limb movement index (PLMI), respectively. Weinreich et al. (26) introduced a new sleep disorder index (SDI), the sum of the AHI and PLMI, and compared the agreement between SleepMinder-derived SDI and PSG-derived SDI. The correlation coefficient of the association between the SDIs generated by SleepMinder and PSG was 0.79, indicating a strong positive correlation. According to Zaffaroni et al. (28) and Weinreich et al. (26), SleepMinder is a viable alternative to conventional methods of detecting OSA and PLMS.

In 2020, Tasi et al. (25) applied a non-contact sensor to monitor the vital signs and body movements of patients with coronavirus disease (COVID-19) in an isolation ward. The non-contact sensor was a non-contact self-injection locking (SIL) radar (SIL Radar Technology Inc., Kaohsiung, Taiwan). The SIL radar was developed to measure vital signs, and it includes the following components: (1) receive antenna, (2) transmit antenna, (3) differential voltage-controlled oscillator with an injection port, (4) frequency demodulator composed of a mixer and a delay line, (5) low-pass filter, (6) bandpass filter, and (7) digital signal processor with built-in analog-to-digital converter and digital-to-analog converter (33). The continuous wave signal emitted from the oscillator embedded in the SIL radar is reflected by the target and injected back into the same oscillator, thereby forming an SIL state (34). The generated signals were modulated by a voltage-controlled oscillator, processed, and analyzed using a digital signal processor (33). The SIL radar can operate at a distance of 4 m from objects and is capable of stable vital sign measurement, even at an operating frequency of 3.6 GHz and an output power of 0 dBm (35). In a study by Tasi et al. (25), an SIL radar was fixed to the ward ceiling to detect a patient’s body temperature and HR. The monitoring results of body temperature and HR from the SIL radar for two COVID-19 patients were compared with the nurses’ records. The *p*-value obtained using Fisher’s exact test was 0.139 for body temperature and 0.292 for HR, indicating that the data collected by the SIL radar and nursing records were not significantly different. Additionally, the SIL radar can detect human face and body movements, allowing for real-time confirmation of patients’ coughing and breathing movements. Coughing and dyspnea are considered significant symptoms of COVID-19. Therefore, the SIL radar allows for continuous monitoring of COVID-19 patients’ conditions, enabling clinicians to save time compared to the conventional method of direct observation. Moreover, continuous health monitoring of patients in nursing stations (clean zones) can reduce the risk of infection.

In 2020, Yoon et al. (27) demonstrated the feasibility of using a non-contact sensor for sleep monitoring compared with PSG in a sample of 10 participants with sleep disorders. The non-contact sleep monitoring device consisted of a passive infrared sensor, a microwave sensor (WAVE, Sharp, Japan), and a smartphone (Galaxy Note 8, Samsung, Korea). The activity of the object was detected by an infrared sensor and recorded every 2 s, whereas the RR and HR were measured every 200 ms by a microwave sensor and then averaged over minutes. Changes in the RR- and HR-related frequencies over time were visualized in graphs and checked using the smartphone. The collected activity, RR, and HR data were used to predict sleep stages and evaluate sleep quality. Sleep was classified into four stages: awake, rapid eye movement (REM), light, and deep sleep. Additionally, the total sleep time, sleep efficiency, and wake after sleep onset (WASO) were used to evaluate sleep quality. Sleep efficiency was defined as the percentage of total time spent sleeping in bed, and WASO was defined as the time spent awake after sleep onset. The accuracy of the non-contact sensors in estimating sleep stages was 98.65%, and a significant positive correlation was found between the non-contact sensors and PSG for evaluating sleep quality (total sleep time, *r* = 0.97; sleep efficiency, *r* = 0.996; WASO, *r* = 0.99).

In 2022, Edanami et al. (24) evaluated the heart-signal detection performance of a non-contact medical radar-based vital sign monitoring system. Their sample included three infants in the neonatal intensive care unit (NICU); an electrocardiogram (ECG) bed monitor (BSM-6301, NIHON KOHDEN Co., Japan) simultaneously measured heart signals with a non-contact sensor. The non-contact sensor included a 24-GHz radar (NJR4262, New Japan Radio Co., Ltd., Japan) and signal acquisition and analysis software. The distance between the radar sensor and the target object was 5 cm, and the radar detected the movement of the object by continuously emitting radio waves of a certain frequency and receiving the reflected waves. Heartbeat peaks were estimated from the heart signal to obtain the interbeat interval (IBI) and heart rate variability (HRV) values. IBI was defined as the time interval between two neighboring heartbeat peaks, and HRV was estimated as a time series of IBI. The agreement between the non-contact radar sensor and ECG for the HR was 99%, and the correlation coefficients of the associations between the non-contact sensor and ECG for the IBI, low-frequency (LF), and high-frequency (HF) HRV were 0.82, 0.98, and 0.95, respectively. These results indicate the excellent performance of radar-based non-contact sensors in analyzing infant heart signals.

## 4 Discussion

This study explored the application of non-contact sensors for health monitoring in hospital settings. Based on the included studies, non-contact sensors are believed to have ample potential for monitoring cardiac and respiratory activities and body movements during sleep (26–28), vital signs of patients in COVID-19 isolation wards (25), and cardiac activity of infants in NICU settings (24). Additionally, monitoring health status using non-contact sensors can alleviate the excessive workload of the nursing staff, and real-time monitoring can help the hospital personnel detect the risk to patients in the ward at any time (25).

Of the five included studies (24–28), four (24–26, 28) used radar-based non-contact sensors, and one (27) used an infrared sensor and a microwave sensor to detect body movement and parameters related to cardiac and respiratory activities. Radar sensors detect the position and velocity of an object by analyzing the signals that radio waves reach and then reflect (36). Infrared sensors determine the presence of an object by detecting the infrared radiation generated by the heat emitted by the object (37). The energy level of the infrared light changes depending on the temperature of the object, allowing it to detect changes in the temperature or position of the object (37, 38). Microwave sensors detect movement by emitting microwaves and receiving signals reflected from objects (39). The operating principles of radar sensors and microwave sensors are rather similar, and when radar sensors and microwave sensors are combined, they are classified as microwave radar sensors (39). Radar and infrared sensors are commonly used as non-contact sensors for monitoring health status, and this technology is becoming more sophisticated with the introduction of algorithms for accurate signal processing and analysis (24). However, the health status that can be monitored using non-contact sensors is limited to vital signs such as HR, RR, blood pressure, body temperature, and oxygen saturation. Blood glucose is often cited as a major concern in health-conscious people (40, 41). The most widely used device for measuring blood glucose is continuous glucose monitoring (CGM), which is an invasive method for checking blood glucose levels by inserting a needle into the skin (11). Although CGM is known to help manage blood glucose profiles, it has been reported to be underutilized by people with diabetes, citing fear of invasive equipment and the hassle of wearing the devices (42, 43). Recently, various sensors such as colorimetric and fluorescent sensors have been developed as wearable sensors for health monitoring (44). Colorimetric and fluorescence sensors can be used to collect and analyze various biochemical information, such as glucose or chloride ion concentration (45–47). The development of a device that can analyze the composition of sweat using non-contact sensors is expected to provide significant benefits for blood glucose management in health-conscious individuals or patients with diabetes. Furthermore, the development of technologies capable of accurately recognizing sleep positions has recently gained interest (48). Movements of the body during sleep are closely correlated with sleep quality and health outcomes (e.g., sleep apnea or sudden infant death syndrome), making them an important factor in health monitoring (49, 50). In 2022, Islam et al. (51) developed a technology using a microwave Doppler radar that could accurately measure cardiopulmonary movement patterns in three sleep positions: supine, side, and prone. Applying this technology in hospital settings, such as the NICU or wards for patients with sleep disorders, could enable the immediate detection of potentially risky situations during sleep, allowing for prompt early intervention.

With the growing interest in non-contact sensors for health monitoring, related technologies are constantly being developed. Many previous studies have reported that non-contact sensors provide more accurate measurements than standard laboratory methods for measuring vital signs (52–54). However, non-contact sensors are not widely used in clinical practice. Despite the abundance of evidence supporting their superior performance, non-contact sensors are yet to become commercially available for several reasons.

First, if the results of a standard measurement tool, which serves as a benchmark for evaluating the accuracy of non-contact sensors, are deemed unreliable, there is a potential risk of misinterpreting the performance of the non-contact sensor. One example is the controversial reliability of PSG, which is the standard method for determining sleep disorders. In 2008, Levendowski et al. (55) reported that high night-to-night variability in OSA could compromise the reliability of PSG results. In 2022, Lee et al. (56) conducted a meta-analysis to evaluate the inter-rater reliability of manual scoring of sleep stages using PSG results. Pooling results from 11 articles, they reported that inter-rater reliability was high for wake (stage W) and REM sleep (stage R), “moderate” for moderate sleep (stage N2) and deep sleep (stage N3), and “fair” for light sleep (stage N1). Overall, the results of the PSG were considered reliable, but the results were poor at certain stages, suggesting that validity needs improvement. These issues should be considered when interpreting the results and evaluating the performance of non-contact sensors.

Second, the measurement performance of non-contact sensors using PPG can vary depending on skin type, sex, or surrounding environment. In 2020, Nowara et al. (57) reported that dark skin types significantly affected the PPG sensor measurement results, with women tending to have a slightly lower measurement accuracy. In 2023, Zhao et al. (58) reported that the non-isothermal nature of the human body can cause discrepancies in readings when non-contact infrared sensors measure the body temperature at a location different from that of a reference device. In addition, blood flow or skin thickness at a particular measurement site can have an impact and environmental factors such as ambient temperature or humidity can act as confounding variables (58, 59). Studies reporting the performance of non-contact sensors have focused on small populations and not on multicenter and multicultural populations. Furthermore, these studies were not designed to consider all human characteristics. Future studies should evaluate the performance of non-contact sensors in diverse healthcare settings and individuals with different characteristics to produce generalizable results.

Third, separating cardiac and respiratory signals perfectly remains a significant challenge. As the amplitude of the heartbeat signal is smaller than that of the respiratory signal, it can be easily distorted by the harmonics of the respiratory signal, necessitating advanced techniques to clearly distinguish between cardiac and respiratory signals (60). In 2023, Uddin et al. (61) developed a system using a microwave Doppler radar that can classify normal breathing, apnea, and hypopnea patterns through HRV-based feature extraction. HRV refers to the variation in time intervals between consecutive heartbeats, and the HF and LF components of HRV exhibit significant changes under different breathing patterns (61). Therefore, future research should consider using HRV as a key biomarker to advance the technologies for separating cardiac and respiratory signals. Another approach is to use ultra-wide band (UWB) sensors. Several previous studies demonstrated that UWB is highly resistant to multipath effects, making it excellent for separating cardiopulmonary signals (62–64).

Fourth, the technology for processing the signals caused by random body movements (RBM) has limitations. During sleep, the signals generated by unexpected RBM are much larger than those obtained from regular chest movements, which can obscure the respiratory signals (65). Previous studies set the maximum amplitude and normal breathing rate standards for respiratory signals based on data collected over a period of time from participants in a static position after falling asleep. Sudden increases in amplitude beyond these standards were classified as unexpected RBM, and signals from these movements were excluded from the analysis (65, 66). However, this signal-processing method is difficult to apply in clinical settings. In the technology development stage, biosignals are generally measured and verified within a defined range of movements of healthy volunteers. However, in clinical settings, patients exhibit various clinical characteristics, including sudden muscle spasms, seizures during sleep, apnea, and hypopnea, making it difficult to predict their sleep behavior patterns. Therefore, future research should consider a wider range of movement characteristics during the development process to enable non-contact sensing technology to learn from diverse output signals. In addition, there is a need for further advancements in techniques to precisely separate the noise caused by movement from respiratory signals.

Finally, concerns regarding privacy violations must be addressed. Sensor devices collect and analyze user biometric information; hence, the risk of violation of personal information cannot be ignored (67). To address this issue, clear and easy-to-understand privacy policies must be published to help users recognize that non-contact sensors pose a low risk of privacy infringement and leakage (68). Additionally, installing non-contact sensors for real-time monitoring increases the risk of users feeling like they are being watched, and they may feel uncomfortable being recorded all day (69). Before applying the device to the user, the personal information to be collected should be disclosed, the monitoring procedures should be explained in detail, and consent must be obtained.

Owing to these technical limitations, current non-contact sensors may be perceived as less reliable. However, in hospital environments, where accurate health monitoring results are expected, a compromise should be sought. We discuss the following trade-off scenario: a large-scale temperature detection system can quickly screen many people, but the accuracy of individual measurements may be lower. In situations where it is crucial to quickly determine the infection status of a large group, even at the expense of individual accuracy, non-contact sensing technology can be adopted. For instance, at the hospital entrance, basic screening and non-contact temperature checks can be used to identify patients with potential COVID-19 symptoms. In this case, minimizing the infection risk holds greater value than achieving a precise diagnosis, thus justifying the use of non-contact sensors. When using non-contact methods to monitor large groups and selectively re-examine those who exceed certain thresholds, several advantages emerge: (1) it significantly reduces the time required compared to measuring each individual with contact-based equipment, (2) it alleviates the excessive workload of healthcare providers, (3) it saves costs related to labor and equipment use, and (4) it alleviates the psychological burden of infection risk for both patients and healthcare providers. By prioritizing infection prevention over absolute reliability, non-contact solutions can be used as the primary health monitoring method to maximize their benefits.

In conclusion, non-contact sensors are expected to be suitable alternatives to contact devices for health monitoring in hospital settings. However, challenges regarding performance enhancement and privacy protection remain unaddressed. In the future, non-contact sensors are expected to overcome these limitations and be widely used for full-cycle health monitoring.

## Author contributions

YJC: Conceptualization, Data curation, Formal analysis, Funding acquisition, Investigation, Methodology, Project administration, Resources, Software, Validation, Visualization, Writing – original draft, Writing – review & editing. GWL: Conceptualization, Data curation, Formal analysis, Funding acquisition, Investigation, Methodology, Project administration, Resources, Validation, Visualization, Writing – original draft, Writing – review & editing. JSM: Conceptualization, Data curation, Formal analysis, Funding acquisition, Investigation, Methodology, Project administration, Resources, Supervision, Validation, Visualization, Writing – original draft, Writing – review & editing. MCC: Conceptualization, Data curation, Formal analysis, Funding acquisition, Investigation, Methodology, Project administration, Resources, Software, Supervision, Validation, Visualization, Writing – original draft, Writing – review & editing.
